# Chlorella Supplementation Reduces Blood Lactate Concentration and Increases O_2_ Pulse during Submaximal and Maximal Cycling in Young Healthy Adults

**DOI:** 10.3390/nu16050697

**Published:** 2024-02-29

**Authors:** Harry White, Tom Gurney

**Affiliations:** Division of Surgery & Interventional Science, University College London, London W1T 7HA, UK; harry.white.19@ucl.ac.uk

**Keywords:** algae, chlorella, cycling, lactate, exercise performance

## Abstract

Chlorella supplementation is reported to improve ${\dot{V}O}_{2\max}$ following extended supplementation periods (~3 weeks). However, there is little research on its impact over submaximal exercise intensities and following shorter supplementation regimens. This study aimed to investigate the efficacy of 6 g/day 2-day chlorella supplementation on exercise performance in healthy young adults. Twenty young healthy adults (Males = 16, Females = 4) (Age 22 ± 6 years, ${\dot{V}O}_{2\max}$ 42.7 ± 9.6 mL/(kg·min)) were recruited for this double-blinded, randomised cross-over study. Participants ingested 6 g/day of chlorella or a placebo for 2 days, with a one-week washout period between trials. Exercise testing consisted of a 20 min submaximal cycle at 40% of their work rate max (WR_max_) (watts), followed by an incremental ${\dot{V}O}_{2\max}$ test. Lactate (mmol/L), heart rate (b/min), oxygen consumption (mL/(kg·min)), O_2_ pulse (mL/beat), respiratory exchange ratio (RER), and WR_max_ were compared across conditions. Following chlorella supplementation, blood lactate levels were significantly lower (*p* < 0.05) during submaximal exercise (3.05 ± 0.92 mmol/L vs. 2.67 ± 0.79 mmol/L) and following ${\dot{V}O}_{2\max}$ tests (12.79 ± 2.61 mmol/L vs. 11.56 ± 3.43 mmol/L). The O_2_ pulse was significantly higher (*p* < 0.05) following chlorella supplementation during submaximal (12.6 ± 3.5 mL/beat vs. 13.1 ± 3.5 mL/beat) and maximal exercise intensity (16.7 ± 4.6 mL/beat vs. 17.2 ± 4.5 mL/beat). No differences existed between conditions for oxygen consumption, RER, ${\dot{V}O}_{2\max}$, or WR_max_. A total of 2 days of 6 g/day chlorella supplementation appears to lower the blood lactate response and increase O_2_ pulse during both submaximal and maximal intensity exercise but did not lead to any improvements in ${\dot{V}O}_{2\max}$.

## 1. Introduction

*Chlorella vulgaris* (chlorella) is a green, unicellular alga used in multiple biopharmaceutical, nutraceutical, and biotechnology industries, but its consumption for functional benefits within humans outside the traditional industries is on the rise [1]. These functional benefits arise from chlorella’s antioxidant, vasodilatory, and anti-inflammatory properties [2,3,4] which may form its potential as a performance-enhancing supplement. Algae consumption is gaining traction, with similar algae such as spirulina garnering more attention in the exercise nutrition field [5,6]. Chlorella has vast multicomponent properties; 43–58% of its dry weight is a complete protein (which includes all the essential and non-essential amino acids) [7]. Chlorella is also an important source of carotenoids (e.g., lutein and β-carotene), photosynthetic pigments, and vitamins (B-Vitamins and niacin) [8].

Initial studies investigating chlorella have focused on potential applications within a clinical remit and reported positive results. Thirteen weeks of chlorella supplementation was shown to suppress the elevation of blood pressure (BP) in spontaneously hypertensive rats [9]. A synergistic action between several components of chlorella was proposed as the mechanism behind this vascular enhancement, with the arginine component suggested to increase the generation of the endothelium-derived relaxing factor, a potent vasodilator. Further studies found that chlorella supplementation reduced blood pressure in mildly hypertensive men along with a reduction in low-density lipoprotein cholesterol [10]. Mechanisms behind the individual micronutrients responsible for this effect on BP were unable to be ascertained. A study on middle-aged men reported that a 6 g/day 28-day chlorella supplementation period decreased arterial stiffness through an increase in nitric oxide (NO) production by the vascular endothelium [2]. Arginine and various antioxidants (β-carotene, vitamin C and E) were suggested as the micronutrients responsible for this vasodilation, and a similar mechanism could be behind the decreases in BP in the studies above. These effects may appeal to athletes; increased peripheral vasodilation enhances blood flow and oxygen delivery to working skeletal muscles. This is beneficial through improving aerobic respiration and ATP production [11], along with increasing the extraction of by-products of metabolic activity following exercises which are known to accelerate fatigue. 

Although limited, studies have aimed to utilise the clinical benefits of chlorella in the exercise nutrition field. Research has shown that a 6 g/day 28-day chlorella supplementation protocol increases ${\dot{V}O}_{2\max}$ in a healthy population [12] and in young men with insufficient micronutrient status [13]. Combined with high-intensity training, 0.9 g/day of chlorella supplementation for 8 weeks also leads to a 73% increase in Peroxisome Proliferator-activated Receptor Gamma Coactivator 1-alpha (PGC-1α) (a known regulator of mitochondrial biogenesis) in overweight women [14]. In a rodent study, chlorella supplementation also led to an improvement in maximal swimming times and a reduction in blood lactate [15]. However, there is scarce research investigating chlorella’s effect on submaximal exercise intensities. Lower exercise intensities are particularly important to cyclists as training and races consist of many intensities. Hence, a supplement that diminishes fatigue at lower exercise intensities and preserves energy levels for higher intensities would be attractive to cyclists. Indeed, a recent study investigated the blood lactate response during 55% WR_max_ cycling following a 6 g/day 21-day chlorella supplementation period, with positive results reported [16]. Yet there is still scope for further research, particularly utilising a shorter loading period. For example, previous research has often necessitated prolonged supplementation periods (14–28 days), demanding participants to ingest large quantities of capsules each day over an extended duration (up to 15 capsules per day for 28 days), which may reduce the appeal to using chlorella as an ergogenic aid. Recently, a single 6 g dose of chlorella was shown to increase plasma concentrations of carotenoids, lutein, and B-carotene [17]. Given that capsules are currently the best ingestion method (to be able to successfully blind participants, but also due to chlorella possessing poor taste and smell), if research can demonstrate that shorter periods of chlorella supplementation can still lead to ergogenic improvements, it would significantly enhance the attractiveness of chlorella as an exercise supplement. However, to the best of our knowledge, whether a short loading period of chlorella will influence exercise performance is still unknown. 

Recognising the limitations and inconsistencies in the previous literature, this study will address the current gap in the literature on chlorella supplementation and exercise performance by employing a supplementation period and exercise intensity which is yet to be investigated whilst keeping the daily dose akin to the previous efficacious literature. Therefore, this study aims to investigate the effect of 6 g/day 2-day chlorella supplementation on submaximal and maximal exercise performance in young healthy adults, through investigating blood lactate levels, heart rate response, O_2_ pulse, and oxygen consumption. 

## 2. Materials and Methods

### 2.1. Participants

**Figure A1:**
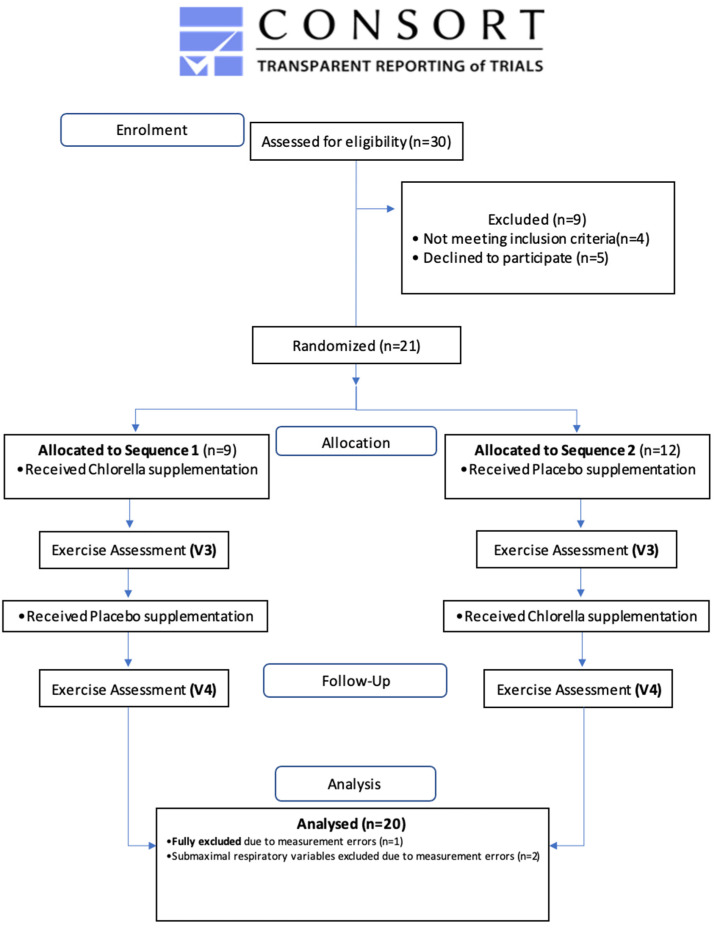
CONSORT flow diagram showing participant flow through each stage of the trial.

Twenty participants (Males = 16, Females = 4) were recruited for this double-blinded, randomised, cross-over study (mean ± SD; Age 22 ± 6 years, Height 174.7 ± 6.7 cm, Mass 72.0 ± 14.0 kg, ${\dot{V}O}_{2\max}$ 42.7 ± 9.6 mL/(kg·min). The CONSORT Flow Diagram (Appendix A, Figure A1) shows the progression of participants from recruitment through to analysis. Participants completed a physical activity readiness questionnaire (PAR-Q) to screen for any risks of exercise and provided written consent for the study. Participants were included if they were aged 18–50 and completed at least 3 h of exercise per week. Any participants with a current injury, history of smoking, cardiovascular disease, any known allergies to mould or algae, or any contraindications to exercise identified in the PARQ were excluded from the study. Participants were asked to not modify their lifestyle, including diet and exercise, throughout the study. Participants were asked to refrain from any alcohol and caffeine consumption or vigorous exercise 24 h before testing, adherence was checked via a 24 h food and training log, adherence was 100%.

### 2.2. Study Design

**Figure 1:**
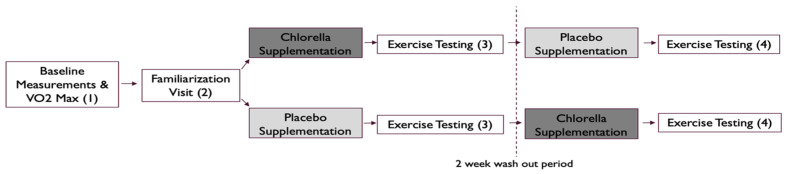
Illustration of the study design.

The first visit consisted of a ${\dot{V}O}_{2\max}$ test which was used to calculate the appropriate load for the future submaximal exercise bouts in visits 2–4. The second visit involved familiarising participants with the exercise protocols that would be used post supplementation (see below). In a randomised cross-over design, participants were then randomly allocated to receive either the placebo or chlorella first by an independent academic member of staff. They then self-administered 12 tablets (6 g) a day postprandial (4 with breakfast, 4 with lunch, and 4 with dinner) for two days. Participants returned to the lab at the exact same time of day, the morning after supplementation finished for post-supplement exercise testing (V3 and V4). This was followed by a minimum one-week washout period and participants then repeated supplementation and exercise testing with the alternate trial assignment. All methods were followed using the PRESENT2020 checklist; a schematic illustration of the study design can be seen in Figure 1.

### 2.3. Chlorella and Placebo Capsules

The placebo (microcrystalline cellulose) and chlorella capsules (Indigo Herbs Limited—see Appendix A, Table A1 for the nutritional breakdown) used in this study were of identical weight and appearance. Indigo Herbs Limited were able to provide a certificate of analysis which detailed compliance with contaminants, quality control, and standardised extraction methods (Supplementary File S1). Both powders were placed into identical opaque capsules and an independent academic member of staff coded the capsules to remove any bias from researchers or participants. In total, 100% of participants reported complete adherence to the supplementation protocol with only one side effect being verbally reported: a ‘grassy taste’ during eructation following chlorella supplementation. Thirteen participants responded to our post-study questionnaire; four felt they could identify which supplement period was chlorella, but only two of those were able to correctly do so.

### 2.4. Familiarization Visit (V1)

During the initial visit, anthropometric measurements were taken. Participants were familiarised with the bike ergometer (Lode Cycle Ergometer, Lode BV, Düsseldorf, Germany), seat height was measured and standardised for future visits. Participants completed an incremental ramp protocol ${\dot{V}O}_{2\max}$ test, establishing their maximal work rate (WR_max_). The test consisted of a two-minute warm up at 75 watts, resistance then continuously increased linearly at 25 watts per minute until participants were unable to maintain a cadence of above 60 revolutions per minute (r/min) for more than 10 s or until volitional exhaustion. The test was followed by two minutes of active recovery at 25 watts. The peak watts measured upon termination of the test were defined as their work rate max, and this value was then used to calculate appropriate 40% WR_max_ resistance for subsequent submaximal exercise bouts.

### 2.5. Exercise Testing (V2, V3, V4)

Resting lactate (Lactate Pro 2, Arkray Factory Inc., Koka, Japan) measurements were taken from a capillary finger prick sample. Respiratory variables, O_2_ pulse (Vyntus CPX, Vyaire Medical GmbH, Höchberg, Germany), and heart rate (HR) (Polar F10, Polar Electro Oy, Kempele, Finland) were measured continuously and averaged over 5 min intervals for data analysis. The submaximal test comprised a minute of stationary rest on the bike to allow all cardiorespiratory variables to normalise, followed by 20 min at 40% WR_max_ (V1). Participants were asked to maintain a cadence of 60–80 r/min and remain seated on the saddle throughout the test. Lactate measurements were taken again at 10 min and upon the test’s immediate completion. After the submaximal test, participants had a 15 min rest period during which they could drink water (which was recorded and standardised for future visits). This was then followed by an incremental ramp protocol ${\dot{V}O}_{2\max}$ test which followed the same protocol used on V1. Blood lactate measurements were taken at 0 min and then upon the test’s termination. All respiratory variables were averaged over 15 s. ${\dot{V}O}_{2\max}$ was determined by the highest ${\dot{V}O}_{2}$ (oxygen consumption) value that was recorded from the 15 s averages prior to test termination. Given that the exercise testing procedures were identical to a previous study in our lab [18], the authors direct the reader to this paper for the test re-test reliability of the variables (coefficient variation %). 

### 2.6. Statistical Analysis 

G*Power (Version 3.1.9.6 for Mac OS X) was used for a priori power calculation. The sample size in this study was enough to detect the effects of supplementation at 80% power, using an α of 5% and a medium effect size (0.5). All data is presented as mean ± SD. IBM SPSS (Version 28.0.1.1) was used to carry out all statistical procedures. *p* values ≤ 0.05 were considered statistically significant. A Shapiro–Wilk test was used to assess normality along with Mauchly’s test of sphericity to establish any potential violations. Any violations identified were corrected using the Greenhouse Geisser. Within subjects, a two-way repeat measures ANOVA was used to analyse HR, lactate, and all respiratory variables during the submaximal test. To adjust for multiple comparisons, a post-hoc Bonferroni correction was used for the family-wise error rate within the ANOVAs to determine any differences. ${\dot{V}O}_{2\max}$, and WR_max_ were compared using a two-tailed paired sample *t*-test. 

## 3. Results

### 3.1. Submaximal Testing 

#### 3.1.1. Lactate

**Figure 2:**
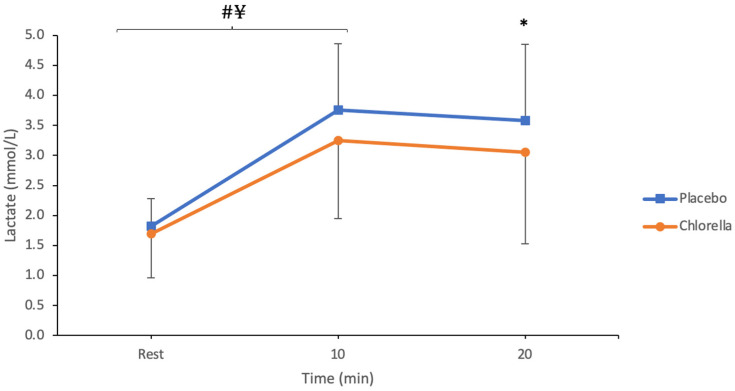
Lactate (mmol/L) during the 20 min submaximal test (N = 20). * Signifies *p* < 0.05 between conditions. # ¥ signifies both chlorella (¥) and the placebo (#) within trial increase *p* < 0.05.

During the 20 min submaximal cycle, the average blood lactate response was lower following chlorella supplementation (2.67 ± 0.79 mmol/L) compared to the placebo (3.05 ± 0.92 mmol/L) (*p* = 0.05, ES = 0.194, Observed Power = 0.528) (Figure 2). A post-hoc analysis revealed lactate levels to be significantly lower between conditions at the 20 min time point (*p* = 0.04); however, the 10 min time point did not reach significance (*p* = 0.062). A significant effect for time was detected (*p* < 0.001) with values at rest being significantly lower than the other time points (*p* < 0.001). There was no difference between 10 and 20 min for both conditions (Chlorella: *p* = 0.40, Placebo: *p* = 1.00). There was no supplement × time interaction identified (*p* > 0.05).

#### 3.1.2. O_2_ Pulse

**Figure 3:**
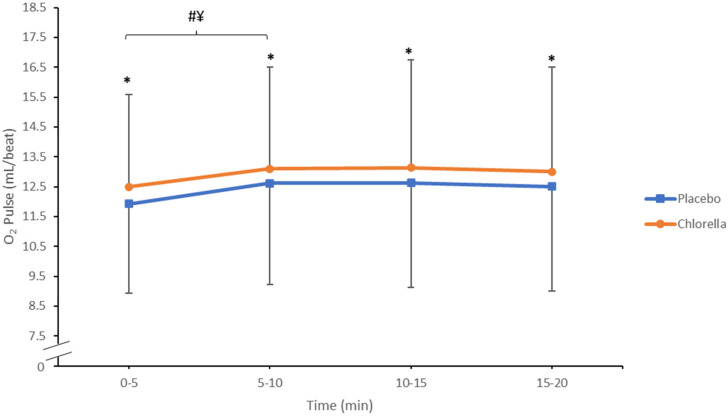
O_2_ pulse (mL/beat) during the 20 min submaximal test (N = 18). * Signifies *p* < 0.05 between conditions at each time point. # ¥ signifies both chlorella (¥) and the placebo (#) within trial increase *p* < 0.05.

The O_2_ pulse was significantly higher following chlorella supplementation (13.1 ± 3.5 mL/beat) compared to the placebo (12.6 ± 3.5 mL/beat) (*p* = 0.005, ES = 0.377 Observed Power = 0.855) (Figure 3). The post-hoc analysis revealed chlorella levels to be significantly higher than the placebo during each 5 min average of the submaximal test (Q1: *p* = 0.01, Q2: *p* = 0.02, Q3: *p* = 0.01, Q4: *p* = 0.01). There was also a significant effect for time detected (*p* < 0.001), with Q1 significantly lower than all other quartiles in both conditions (*p* < 0.027). There was no supplement × time interaction identified (*p* = 0.92). Due to a measurement error during the submaximal test, two participants had their submaximal VO_2_ data excluded. 

#### 3.1.3. Heart Rate 

On average, HR was marginally lower throughout the 20 min cycle following chlorella supplementation (131 ± 13 b/min) compared to the placebo (134 ± 15 b/min); however, this did not reach statistical significance (*p* = 0.07). A significant effect for time was detected (*p* < 0.001), with pairwise comparisons reporting HR to significantly increase between each quartile following chlorella supplementation (*p* < 0.05). The placebo also displayed the same effect, however there was no significant increase between Q2 and Q3 (*p* > 0.05). There was no supplement × time interaction identified (*p* = 0.67). 

There was no significant effect of supplementation on VO_2_ (chlorella = 24.1 ± 4.5 mL/(kg·min) vs. placebo = 23.8 ± 4.0 mL/(kg·min), *p* = 0.27) or RER (chlorella = 0.94 ± 0.03 vs. placebo = 0.95 ± 0.04, *p* = 0.60), and there was no condition*time interaction for either of these variables during the submaximal cycle (*p* = 0.89 and *p* = 0.10, respectively).

### 3.2. Incremental Test to Fatigue

**Figure 4:**
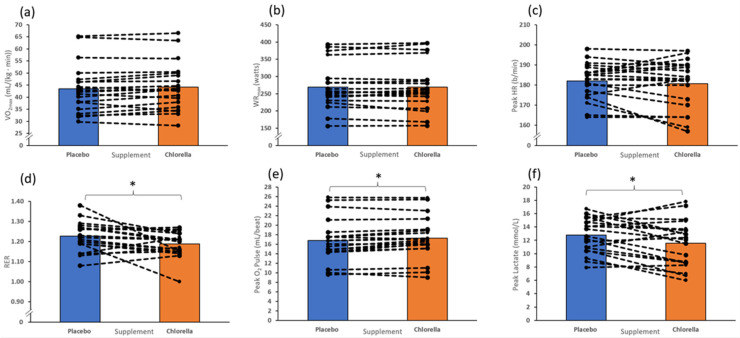
Incremental test to fatigue variables following the 20 min submaximal cycle after a 2-day 6 g/day supplementation of either chlorella or the placebo (N = 20). (**a**) ${\dot{V}O}_{2\max}$, (**b**): WR_max_, (**c**) peak HR), (**d**) RER, (**e**) peak O_2_ pulse, (**f**) peak lactate. * indicates *p* < 0.05.

During the incremental test to fatigue, peak RER (*p* = 0.03, 95% CI = 0.01–0.07), O_2_ pulse (*p* = 0.01, 95% CI = −0.87–−0.11), and lactate (*p* = 0.02, 95% CI = 0.21–2.25) were all significantly different between conditions. However, no differences were found between ${\dot{V}O}_{2\max}$ (*p* = 0.13, 95% CI = −1.64–0.23)_,_ WR_max_ (*p* = 0.75, 95% CI = −4.02–5.52), and peak HR (*p* = 0.35, 95% CI = −1.75–4.70), see Figure 4.

## 4. Discussion

To the best of our knowledge, this is the first study to investigate the influence of chlorella supplementation following a short 2-day supplementation period on submaximal and maximal cycling intensities. The results indicate that a 2-day 6 g/day chlorella supplementation period does not provide any performance benefits during ${\dot{V}O}_{2\max}$ but does lower the blood lactate response. This can similarly be observed during the submaximal cycle (Figure 2), with the O_2_ pulse being higher after chlorella supplementation (Figure 3). Collectively, the results from this study both conflict and support the previous literature; we scrutinise why below.

### 4.1. Submaximal Exercise 

#### 4.1.1. Lactate

This study found that chlorella supplementation lowered blood lactate levels during submaximal cycling, see Figure 2, with significant reductions observed at the 20 min time point. Although lactate levels were lower at the 10 min interval, this did not reach statistical significance. Thirteen of the twenty participants had a lower blood lactate response following chlorella supplementation, with two participants having no change in blood lactate response. Lactate was initially thought to be a toxic metabolite of exercise; however, it is now perceived as an essential multifunctional signalling molecule affecting various cells and tissues throughout the body [19]. Lower lactate levels during submaximal exercise are indicative of a higher tolerance to exercise due to decreased homeostatic disturbance [20], which therefore may delay the onset of fatigue. 

In agreement with our findings, a recent study investigating the effect of 21 days of 6 g/day chlorella supplementation found a decrease in the blood lactate response. However, this study utilised a higher intensity (55% WR_max_) and a longer submaximal cycle (1 h). Supporting our findings on lactate metabolism, a rodent study reported that combining high-intensity intermittent exercise with 6-week chlorella supplementation influenced exercise performance through changes in muscle glycolytic and oxidative metabolism [21]. This combined intervention elevated Monocarboxylate Transporter 1 (MCT1) and PGC-1α protein levels in red muscle. PGC-1α regulates the expression of MCT1; this monocarboxylate transporter favours the uptake of lactate into working skeletal muscles [22] and is the proposed activity behind reduced serum lactate levels observed in the rodent study. It has also been demonstrated that muscle-specific over expression of PGC-1α increases transcriptional regulation of the lactate dehydrogenase (LDH) gene and LDH enzyme activity [23]. Activation of muscle LDH decreases blood lactate levels through catalysing the conversion of lactate to pyruvate [24]. While rodent studies can provide insights into biological mechanisms, it is important to exercise caution when extrapolating these findings into a human population, especially following such a short supplementation period. Despite this, to the best of our knowledge, there is one human study which supports the notion that chlorella supplementation (0.9 g/day for 8 weeks) combined with training can induce increases in PGC-1α [14]. 

With respect to the micronutrients found in chlorella that may be responsible for supporting increases in PGC-1α, l-arginine supplementation has been shown to stimulate the expression of PGC-1α, via an increase in NO levels [25]. Indeed, although chlorella contains l-arginine (Appendix A, Table A1), and therefore a similar mechanism may be present in our study, it is important to highlight the dose comparisons of previous studies. For example, in a prior study investigating l-arginine’s effect on exercise-induced serum lactate levels, participants received 5 g per day of l-arginine over a 4-week loading period [26]. The chlorella dosage used in this study, according to Appendix A, Table A1, only contained 198 mg of arginine per day; therefore, another potential mechanistic avenue may need to be explored.

The practical relevance of a small decline in blood lactate levels during exercise is pivotal for both improved exercise performance and enhanced recovery. Firstly, blood lactate concentration has been shown to reflect muscle metabolism [27], and physiological adaptations to the lactate-related fatigue mechanism have been suggested to improve aerobic capacity. This mechanism could be responsible for chlorella’s influence on aerobic endurance in previous studies [12,13]. Secondly, lower lactate levels during exercise could suggest that the body can facilitate the removal of lactate more effectively, leading to faster recovery times between exercise bouts. This would be beneficial for athletes who perform multiple training sessions within a short period. Considering this study was applied in nature, investigating the precise mechanisms of action behind reductions in blood lactate levels was beyond this study’s scope. Further studies are needed in humans, with greater focus on identifying the mechanisms behind chlorella’s influence on the blood lactate response to exercise. 

#### 4.1.2. O_2_ Pulse 

One of the principal findings of this novel study was that O_2_ pulse significantly increased throughout the submaximal cycle following chlorella supplementation, see Figure 3. O_2_ pulse is a cardiopulmonary variable which receives little attention in the exercise testing field. Defined as oxygen uptake relative to HR, it has been proven to be an indirect indicator of stroke volume, particularly at low intensities [28]. A higher O_2_ pulse is indicative of higher exercise tolerance [29], as theoretically, more oxygen can be extracted from the blood into skeletal muscles for the oxidative phosphorylation pathway. However, once again, the multicomponent properties of chlorella result in mechanistic actions which are difficult to construe. Mechanisms resulting in an increased O_2_ pulse will be through either a decrease in HR, increased ${\dot{V}O}_{2}$, or a combination of the two. The results from the submaximal test show that there was both an increase in ${\dot{V}O}_{2}$ along with a marginal decrease in HR throughout the test duration, although this did not reach statistical significance. Following 21 days of 6 g chlorella supplementation, no significant decrease in ${\dot{V}O}_{2}$ was found during a one-hour submaximal endurance cycle [16]; this finding is consistent with our study. Conversely, a 6 g/day 7-day supplementation of a similar alga (spirulina) decreased ${\dot{V}O}_{2}$ during upper body arm cycling [30]. This highlighted the alga as a potential ergogenic aid during arm cycling by reducing the respiratory demand of exercise. Similarly, in this study, a reduction in HR was also observed. At maximal intensity, a higher ${\dot{V}O}_{2}$ is indicative of improved physical conditioning and cardiovascular fitness [31]. However, at submaximal exercise intensities, a higher ${\dot{V}O}_{2\max}$ could be an adversity for exercise performance. This is because working muscles require more oxygen to respire aerobically for the same level of performance. This leads to a higher respiratory demand and increased ${\dot{V}O}_{2}$. Therefore, with regards to exercise performance, ${\dot{V}O}_{2}$ can be an indicator of improved or reduced aerobic capacity depending on the exercise intensity and needs to be interpreted carefully.

Vasodilation is a speculated mechanism behind the increased O_2_ pulse; it is important to note we did not measure vasodilation directly in this study. Vasodilation will reduce peripheral resistance and therefore increase stroke volume and cardiac output [32]. Chlorella supplementation has been shown to decrease arterial stiffness via a proposed increase in NO production in middle-aged individuals [2]. Reduced arterial stiffness results in improved blood flow and consequently aids the clearance of deleterious metabolites associated with metabolic acidosis. This delays the onset of fatigue caused by these metabolites and allows submaximal exercise to be completed with less homeostatic disturbance. Rodent studies have demonstrated that chlorella supplementation can induce dose-dependent relaxation in thoracic aortas, inhibited by NO antagonists [33]. This suggests that the vasodilatory effect is through chlorella interacting with the endothelial-NO pathway, with arginine as the main component responsible for increased NO levels. However, there is uncertainty as to whether l-arginine supplementation elevates NO levels in healthy subjects [34,35]; therefore, another mechanistic action may be at play for the vasodilatory effect. Magnesium has been shown to modulate endothelium-dependent vasodilation [36] as well as many cardiac variables including HR and systemic vascular resistance [37,38]. Chlorella-derived products contain high levels of magnesium (21.6 mg per 6 g), so this could be a future avenue to explore in confirming one of chlorella’s mechanisms of action. 

High bioavailable constituents, such as carotenoids (lutein, β-Carotene, and zeaxanthin) are another component with the potential to influence vasorelaxation [39]. Endothelial dysfunction is exacerbated by increases in oxidative stress and inflammation. This dysfunction reduces NO bioavailability through the rapid oxidative inactivation of NO by excess superoxide (O_2_) [40]. High concentrations of β-carotene are associated with a significant increase in NO levels and bioavailability. Increased release of NO leads to a downregulation of the expression of NF-kB-dependent adhesion molecules in endothelial cells [41]. These adhesion molecules regulate vascular inflammation and therefore downregulation can ameliorate inflammation and reduce endothelial dysfunction. The maintenance of endothelial NO bioavailability is therefore considered beneficial to endothelial functions and in general to vascular health. Previously, a single 6 g dose of chlorella vulgaris has been shown to increase plasma concentrations of β-carotene and other carotenoids [17]. Considering the aforementioned possible benefits of carotenoids on the vasculature, it is therefore plausible to suggest that such a short supplementation period in this study can indirectly elicit improvements in O_2_ pulse and lactate metabolism. Further research is needed within chlorella supplementation to confirm some of these possible vasodilatory effects and how these may impact exercise performance. However, initial results from this study are promising. 

#### 4.1.3. Heart Rate 

The HR response was marginally lower following chlorella supplementation (131 ± 13 b/min) compared to the placebo (134 ± 15 b/min); 13 of 20 participants demonstrated a reduced HR response throughout the duration of the submaximal test, yet this did not reach significance. This finding conflicts with a previous study which demonstrated that 21 days of 6 g chlorella resulted in a significant decrease in HR during a one-hour submaximal endurance test [16]. A potential reason for the disparity between results is that our supplementation period was too short to elicit a significant change during the submaximal cycle. Spirulina, a similar microalga, exhibited a lower HR response and cardiovascular demand during a one-hour submaximal endurance cycle [42]. It was proposed that the increased haemoglobin levels produced an ergogenic effect due to their pivotal role in transporting oxygen from the lungs to working muscles, supporting the oxidative phosphorylation pathway. Another potential reason for the discrepancy with previous studies is due to our limited sample size. Our data showed that following chlorella supplementation, the heart rate was lower throughout all quartiles of the submaximal test; however, it was marginally insignificant (*p* = 0.07). 

l-Arginine has been demonstrated to augment cardiac vagal control in healthy individuals [43], along with exhibiting a negative chronotropic effect in patients with chronic heart failure [44]. The proposed mechanism was l-arginine infusion acting on the NO-l-arginine pathway, which attenuated the HR response to sympathetic activation. Similar mechanisms may be responsible for the physiological changes in this study; however, as previously mentioned, the acute loading period makes this unlikely and future research may need to be directed at some of chlorella’s other micronutrients. As opposed to a direct decrease in HR following chlorella supplementation, alternative reasoning could be the association with the blood lactate response between conditions. Higher lactate levels observed following a placebo indicate metabolic acidosis [45]. The cardiovascular response to this imbalance is an increase in HR to increase lactate shuttling, reduce blood lactate levels, and lower homeostatic disturbances from exercise [46]. This is a potential rationale behind the differences in HR throughout the exercise test between conditions. It is important not to analyse physiological variables in isolation and to appreciate the synergistic effect between variables. This is highlighted above in the relationship between blood lactate and HR during exercise, with chlorella potentially providing a positive response to both variables. However, the authors have limited comprehension as to why the change in lactate and O_2_ pulse were not accompanied by significant changes in HR and VO_2_. It may be due to too much variability in our HR (~±13 b/min) and VO_2_ (~±4.25 mL/(kg·min)) data, as physiologically one would expect the HR response to be lower when lower blood lactate responses were observed. 

#### 4.1.4. ${\dot{V}O}_{2\max}$

The results from this study indicate that 2-day 6/day chlorella supplementation increased peak O_2_ pulse and decreased peak blood lactate concentration. However, this did not correlate to an ergogenic improvement as there was no significant increase in ${\dot{V}O}_{2\max}$ or WR_max._ This contrasts with previous studies which have demonstrated a significant increase in both ${\dot{V}O}_{2\max}$ and WR_max_ following 28 days of chlorella supplementation [12,15]. 

It was hypothesised in this aforementioned study that branched-chain amino acids in chlorella were potentially responsible for the improvement in aerobic performance. Mechanistic pathways proposed for this improvement were an increase in vasodilation, enhancing blood flow to, and excretion of by-products away from metabolising tissue. The increased O_2_ pulse and decreased blood lactate were consistent with this potential mechanism, but 2 days of supplementation was insufficient to produce physiological enhancements observed in previous studies. Further studies should employ a longer dosage period to investigate if there is a minimum duration or dosage of supplementation required to elicit significant improvements in aerobic performance through ${\dot{V}O}_{2\max}$ and WR_max_. This previous study also postulated that improvements in performance were due to nutritional deficiencies in the participants, and comparable results may not be produced in a healthy, nutritionally sufficient population [13]. Therefore, it could be possible that our participants had no overt nutritional deficiency, so chlorella exerted no physiological improvements. However, the improvements in cardiological and serum measures of exercise performance indicate that this theory is unlikely. A high percentage of participants were undergraduate students living away from home, a population which has been shown to have a high proportion of nutritional deficits [47]. Future studies are warranted with a greater focus on the relationship of nutritional status to the efficacy of chlorella supplementation. 

#### 4.1.5. Limitations 

The results from this investigation should be interpreted with caution due to limitations within the study. Primarily, the novelty of our investigation (6 g/day for 2 days of chlorella supplementation on submaximal and maximal cycling intensities) means that there is nothing to directly compare our results to; only vague inferences/speculations can be made with relation to other algae, rodent studies, and other exercise intensities/modalities. Additionally, although we ensured participants were healthy using a PAR-Q to identify health conditions, no measure of participants’ nutritional status was made. This is important because currently, chlorella supplementation has been shown to be more effective in individuals with an overt nutritional deficiency. Therefore, ergogenic improvements may be simply due to nutritional improvement and equivalent results may not be reproduced in individuals without any overt nutritional deficiencies. This is explicitly relevant in our study due to the high proportion of students (95%) comprising our population, with students experiencing high occurrences of insufficient nutrient intake. Furthermore, our study population did not consist of amateur/professional cyclists so are unlikely to take chlorella supplementation to improve their cycling performance; this therefore limits the generalisability of our findings. This is a potential avenue that future research on chlorella supplementation should be directed towards, investigating the effects of chlorella supplementation in elite-level athletes. Another study limitation was that the chlorella powder used was not individually batch-tested for nutritional analysis; therefore, the levels of individual micronutrients within the capsule are unknown. Consequently, with any physiological changes attributed to a specific micronutrient, it is impossible to ascertain the quantity responsible for this change. Further research is needed in this field with greater consideration of nutritional status and the exact contents of chlorella used to produce more reliable results. 

## 5. Conclusions

The novel findings of this study indicate that 2 days of 6 g/day chlorella supplementation significantly reduces the blood lactate response and increases O_2_ pulse during a 20 min submaximal cycle and ${\dot{V}O}_{2\max}$. However, 2 days of supplementation were insufficient to improve ${\dot{V}O}_{2\max}$ and WR_max_. Although this study lacks mechanisms to support our findings, these novel applied findings contribute to the ever-growing body of literature on chlorella’s potential use as a supplement for exercise performance. Future research should aim to investigate chlorella supplementation in an athlete population to see if comparable results are reproduced. A greater focus should also be given to investigating mechanisms behind the changes in the blood lactate response and O_2_ pulse.

## Data Availability

The data supporting the conclusions of this article will be made available by the authors. The dataset is available on request from the authors.

## Supplementary Materials

The following supporting information can be downloaded at: https://www.mdpi.com/article/10.3390/nu16050697/s1.

## Appendix A

**Table A1:** Nutritional contents of chlorella vulgaris capsules used (6 g and 100 g).

	Per 100 g	Per 6 g
Energy KJ/Kcal	1448 KJ/343 Kcal	87 KJ/21 Kcal
Fat	7.8 g	0.47 g
of which saturates	3.1 g	0.19 g
Carbohydrate	6.9 g	0.42 g
of which sugars	1.6 g	0.10 g
Protein	61.3 g	3.68 g
Dietary Fibre	16.1 g	0.97 g
Salt	0.8 mg	0.05 mg
Vitamin E	8.9 mg	0.53 mg
Vitamin B1	1.29 mg	0.08 mg
Vitamin B2	3.1 mg	0.19 mg
Vitamin B3	59 mg	3.54 mg
Vitamin B6	0.96 mg	0.06 mg
Folate	2.3 mg	0.14 mg
Biotin	0.1 mg	0.01 mg
Potassium	882.5 mg	52.95 mg
Calcium	330 mg	19.8 mg
Phosphorus	1210 mg	72.6 mg
Magnesium	360 mg	21.6 mg
Iron	210 mg	12.6 mg
Zinc	69.8 mg	4.19 mg
Carotenoids	945 mg	56.7 mg
Beta-carotene	229 mg	13.74 mg
Chlorophyll	3790 mg	227.4 mg
Chlorophyll-a	1749 mg	104.94 mg
Leucine	4700 mg	282 mg
Arginine	3300 mg	198 mg
Lysine	3000 mg	180 mg
