# Supplementary material for: Chlorella Supplementation Reduces Blood Lactate Concentration and Increases O2 Pulse during Submaximal and Maximal Cycling in Young Healthy Adults
Source: Nutrients. 2024 Feb 29;16(5):697. doi: 10.3390/nu16050697 (PMC10934562; doi:10.3390/nu16050697)
Supplement: Supplementary file 1 [file nutrients-16-00697-s001.zip › nutrients-2869176-supplementary.pdf]

Indigo Herbs
GLASTONBURY
health · nutrition · wellness

Indigo Herbs Ltd
Dyehouse Lane Trading Estate
Glastonbury
England
BA6 9LZ
(+44) 01458 831 447
sales@indigo-herbs.co.uk
www.indigo-herbs.co.uk

This product was packed at the above address and is certified as Organic by:

Organic Food Federation

SERIAL No: 01224/12 GB-ORG-04

Organic Food Federation serial No: 01224/001/12

Product: Organic Chlorella Tablets

General:

Country of origin	China
Storage conditions	Cool, dark, dry conditions away from direct light
Appearance	Characteristic green tablets
Taste	Typical
Moisture	<7%

Heavy Metals Batch Results:

Parameter	Result	Status
Lead (Pb)	0.12	Compliant
Cadmium (Cd)	0.01	Compliant
Mercury (Hg)	0..04	Compliant
Arsenic (As)	0.35	Compliant

Microbiological:

Parameter	Result	Status
Aerobic colony count	20000	Compliant
Presumptive Enterobacteriaceae	<10	Compliant
Presumptive Coliforms	<10	Compliant
E.coli	<10	Compliant
Staphylococcus Aureus	<20	Compliant
Mould	<200	Compliant

Indigo Herbs Ltd
Dyehouse Lane Trading Estate
Glastonbury
England
BA6 9LZ
(+44) 01458 831 447
sales@indigo-herbs.co.uk
www.indigo-herbs.co.uk

Bacillus cereus	520	Compliant
Presumptive clostridium	30	Compliant
Salmonella in 25gr	Not Detected	Compliant
Listeria spp in 25gr	Not Detected	Compliant
Yeast	<200	Compliant

Pesticides:

Parameter	Status
Compliant with Reg (EC) No 396/2005 on pesticide MRL's and BNN Orientation Values	Compliant

This statement is a copy of laboratory results received from our supplier.

Claire Kelly
Quality Manager
Indigo Herbs

Quality Assured by Indigo Herbs means:

- Our products are clearly named & correctly botanically identified (including the Latin name)
- Our products inform clearly where the botanical material is from, and/or where it is manufactured.
- Our products inform clearly how the botanical materials were produced & preserved where possible.
- Our products inform clearly the shelf life and best before dates for the botanical material to remain fresh, potent & nutritionally stable.
- Our products are packed & sealed in re-sealable gold foil or brown paper/foil air tight packaging for loose materials, and pharmacoepial grade brown glass bottles for liquids. This is to ensure optimum storage conditions for an extensive shelf life.
- Our products have full traceability. From the batch number we can trace & recall if necessary the full details & origin of all the ingredients a product contains.
- Our ingredients come to us with a certificate of analysis where possible, demonstrating that they are tested for appearance, taste, odour, constituents, microbiology levels, heavy metals & pesticides.
- Our ingredients meet pharmacoepial grade standards where appropriate.
- All our products are 100% pure botanical materials with absolutely nothing added. (No hidden bulking agents)
- Our production facility is fully compliant with Mendip District council's Food Safety, and Health & Safety standards.

Indigo Herbs Ltd
Dyehouse Lane Trading Estate
Glastonbury
England
BA6 9LZ
(+44) 01458 831 447
sales@indigo-herbs.co.uk
www.indigo-herbs.co.uk

- Our products contain ingredients from suppliers who are fully compliant with their Environmental health departments, and who follow GMP. (Good Manufacturing Practises)
- Our Organic products are certified by The Organic Food Federation GB-ORG-04. These policies are based on the European standards – EU council regulations 834/2007 & 889/2008.
- We have three graphics to demonstrate our quality standards: quality, organic and wild-crafted.
